# Intra-islet glucagon secretion and action in the regulation of glucose homeostasis

**DOI:** 10.3389/fphys.2012.00485

**Published:** 2013-01-03

**Authors:** Qinghua Wang, Xinyun Liang, Susanne Wang

**Affiliations:** ^1^Division of Endocrinology and Metabolism, The Keenan Research Centre in the Li Ka Shing Knowledge Institute, St. Michael's HospitalToronto, ON, Canada; ^2^Department of Physiology, University of TorontoToronto, ON, Canada; ^3^Department of Medicine, University of TorontoToronto, ON, Canada

**Keywords:** glucagon secretion, insulin secretion, β-cells, α-cells, hepatic glucose production

## Abstract

Glucagon, a key hormone in the regulation of glucose homeostasis, acts as a counter-regulatory hormone to insulin by promoting hepatic glucose output. Under normal conditions, insulin and glucagon operate in concert to maintain the glucose level within a narrow physiological range. In diabetes, however, while insulin secretion or action is insufficient, the production and secretion of glucagon are excessive, contributing to the development of diabetic hyperglycemia. Within an islet, intra-islet insulin, in cooperation with intra-islet GABA, suppresses glucagon secretion via direct modulation of α-cell intracellular signaling pathways involving Akt activation, GABA receptor phosphorylation and the receptor plasma membrane translocation, while intra-islet glucagon plays an important role in modulating β-cell function and insulin secretion. Defects in the insulin-glucagon fine-tuning machinery may result in β-cell glucose incompetence, leading to unsuppressed glucagon secretion and subsequent hyperglycemia, which often occur under extreme conditions of glucose influx or efflux. Therefore, deciphering the precise molecular mechanisms underlying glucagon secretion and action will facilitate our understanding of glucagon physiology, in particular, its role in regulating islet β-cell function, and hence the mechanisms behind glucose homeostasis.

## Background

Glucagon is a 29-amino acid peptide synthesized and released from the pancreatic α-cells, where it is produced through the cleavage of proglucagon by prohormone convertase 2 (Rouille et al., 1997; Jiang and Zhang, 2003). Glucagon exerts its biological actions through the activation of glucagon receptors (Gcgr), which is a G protein-coupled receptor found in various parts of the body including the liver, kidney, adipose tissue, brain, and the pancreatic β-cells (Burcelin et al., 1995). Activation of glucagon receptor, particularly in the liver, is a critical determinant in controlling the rate of gluconeogenesis and glycogenolysis (Unger, 1985; Lefebvre, 1995). The production and secretion of glucagon, which enhance hepatic glucose production, are important mechanisms by which the body prevents hypoglycemia (Unger, 1985; Lefebvre, 1995). In both animals (Myers et al., 1991; Young et al., 1993) and humans (Lins et al., 1983; Freychet et al., 1988; Hvidberg et al., 1994), the administration of exogenous glucagon elevates glucose levels during a fasted or fed state. Therefore, glucagon is an effective therapy for treating severe hypoglycemia in humans (Freychet et al., 1988; Carstens and Andersen, 1994; Haymond and Schreiner, 2001; Kedia, 2011).

Postprandial hyperglycemia stimulates insulin secretion, which acts on the liver to suppress glucose production. Concomitantly, increased blood glucose levels suppress glucagon secretion and reduce its stimulatory effects on hepatic glucose production. During insulin-induced hypoglycemia, glucagon raises blood glucose levels by enhancing glycogenolysis and gluconeogenesis (Bansal and Wang, 2008) to restore normoglycemia (Freychet et al., 1988). The maintenance of normoglycemia relies on the body's response to a change in the insulin-to-glucagon ratio. During a fed state, an increase in the ratio promotes the transport of excess glucose from the blood to tissues in order to prevent postprandial hyperglycemia (Jiang and Zhang, 2003). During a fasted state, a decrease in glucose levels stimulates hepatic glucose output, preventing hypoglycemia (Unger, 1985).

Excessive secretion of glucagon contributes to the development of diabetic hyperglycemia as a consequence of increased hepatic glucose production (Sherwin et al., 1976; Unger, 1978; Barg et al., 2000; Gastaldelli et al., 2000). Preclinical studies demonstrated that chronic glucagon perfusion resulted in hyperglycemia, glomerular abnormalities, and impaired glucose tolerance, which are symptoms of early stage type 2 diabetes (Li et al., 2008). Interestingly, acute infusion of glucagon resulted in sustained hyperglycemia as a consequence of stimulated gluconeogenesis and glycogenolysis in the liver (Gastaldelli et al., 2000; Shah et al., 2000), which, however, occurred only in human diabetic subjects with impaired β-cell function, but not in non-diabetic subjects (Rizza et al., 1979). Interestingly, in insulin-withdrawn diabetic patients, the glycemic response to hyperglucagonemia is much greater than those in non-diabetic controls (Sherwin et al., 1976), suggesting that in diabetic individuals, abnormalities in the secretion of not only glucagon, but also insulin, along with the alteration of the glucagon-to-insulin ratio, lead to hyperglucagonemia (Bansal and Wang, 2008; Unger and Orci, 2010).

Since insulin is a physiological suppressor of glucagon secretion (Bansal and Wang, 2008), relative insufficient insulin production or declined insulin action in type 2 diabetes may reduce intra-islet insulin action on the suppression of glucagon secretion from the α-cells (Greenbaum et al., 1991). At the cellular and molecular levels, we have demonstrated that chronic exposure of α-cells to high concentrations of glucose and insulin might impose insulin resistance on these cells (Xu et al., 2006). Insulin resistance in α-cells may result in concurrently unsuppressed glucagon secretion under insulin-stimulatory conditions (Bansal and Wang, 2008; Yan et al., 2009). Thus, in type 2 diabetes, despite the β-cells working at maximum capacity to produce and secrete insulin, hyperglucagonemia, and hyperglycemia persist due to insulin resistance in the α-cells (Xu et al., 2006).

Hypoglycemia can produce a variety of detrimental complications, and sometimes with fatal outcomes. This is a considerable challenge in insulin therapy as failure of glucagon counterregulation during hypoglycemia is a key factor limiting insulin treatment in patients with diabetes (Bolli, 1998; Cryer et al., 2003). Since the brain relies on glucose as its sole source of energy, profound hypoglycemia can cause permanent neurological damage, leading to functional brain failure, and even brain death (Cryer, 2007). It should be noted that, during exogenous insulin therapy, insulin levels do not decrease when glucose levels fall. Persistent suppression of α-cell response may result in defective glucose counterregulation, leading to insulin-induced hypoglycemia, as a consequence of lacking hepatic glucose production under glucagon-stimulated conditions. Iatrogenic hypoglycemia is usually associated with recurrent morbidity in most patients with type 1 diabetes and many with type 2 diabetes (Bolli, 1998; Cryer et al., 2003). Mechanistically, during insulin therapy for type 1 diabetes, the exogenous level of insulin does not decrease in response to the lowering of blood glucose, due to a lack of endogenous glucose-sensing mechanisms (Stutzer et al., 2012). In type 2 diabetes, however, chronic intra-islet hyperinsulinemia may cause α-cell resistance (Tsuchiyama et al., 2007). Presumably, under insulin-stimulatory conditions, intra-islet insulin action may not be able to efficiently exert suppressive effects on α-cell secretion, whereas under glucagon-stimulatory conditions, intra-islet hyperinsulinemia prevents the glucagon response to hypoglycemia (Banarer et al., 2002). Therefore, persistent suppression of glucagon secretion by exogenous insulin impairs the insulin-glucagon fine-tuning system that predominantly maintains glucose homeostasis, and may result in iatrogenic hypoglycemia (Bolli, 1998; Cryer et al., 2003). Particularly, in type 1 diabetes, the lack of insulin-secreting β-cells diminishes the suppressive effect of endogenous insulin on α-cell secretion. As a consequence, the α-cells may become more sensitive to exogenous insulin (Bansal and Wang, 2008).

## Mechanisms of glucagon secretion

Glucose is a predominant factor limiting glucagon secretion (Gerich et al., 1974; Maruyama et al., 1984; Matsuda et al., 2002). The insulin-producing β-cells and the glucagon-secreting α-cells are each featured with a unique set of ion channels (Kanno et al., 2002; Leung et al., 2005) and are both electrically excitable. The two types of islet cells work in a coordinated manner such that an “on-off” mechanism is established to fine-tune the secretion of insulin and glucagon, and insulin-glucagon levels. Typically, under insulin-stimulatory glucose concentrations, β-cells are electrically excitatory, and the α-cells are electrically silent (Gopel et al., 2000). Within the islets, the regulation of glucagon secretion by glucose and paracrine factors (i.e., the β-cell secretory products) is mediated by electrical machinery comprising of a variety of ion channels that determine depolarization or hyperpolarization of the α-cells (Gopel et al., 2000; Cejvan et al., 2003; Gromada and Rorsman, 2004; Gromada et al., 2004; Yan et al., 2009). The secretion of glucagon requires a full depolarization cascade involving the sequential activation of a number of ion channels. Particularly, the activation of T-type Ca^2+^ channels depolarizes the cell to an intermediate membrane potential which activates the tetrodotoxin (TTX)-sensitive Na^+^ channels; the influx of Na^+^ ions further depolarizes the α-cell, leading to the activation of the L- or N-type Ca^2+^ channels and the generation of sustained Ca^2+^ influx, triggering glucagon granule exocytosis. The hyperpolarization-activated cyclic nucleotide-gated (HCN) channels, expressed in the α-cells, are presumably involved in initiating the depolarization cascades (Zhang et al., 2008). Maintenance of the ATP-sensitive K^+^ (K_ATP_) channel activity in α-cells within an appropriate range is critical for allowing the operation of this machinery (Bansal and Wang, 2008). At high glucose concentrations, the closure of the K_ATP_ channels, as a consequence of increased intracellular ATP/ADP ratio, depolarizes the α-cell membrane potential beyond the narrow window, causing voltage inactivation of the depolarization cascade (Bansal and Wang, 2008). At low glucose concentrations, however, the opening of K_ATP_ channels only occurs in a subpopulation of these channels on the α-cell and sets the membrane potential to a very negative value, causing the activation of the T-type Ca^2+^ channels and/or the HCN channels to trigger the subsequent depolarization cascades (Gopel et al., 2000; MacDonald et al., 2007). Importantly, under insulin-stimulatory conditions, insulin appears to be predominant in suppressing glucagon secretion, presumably through either direct interference of the K_ATP_ channel activity, or the α-cell membrane potential—which is exemplified by the insulin-induced activation of the type A receptor for γ-Aminobutyric acid (GABA_A_R)—causing membrane hyperpolarization, or the suppression of the glucagon gene (Bansal and Wang, 2008). Insulin, in cooperation with GABA, suppresses glucagon secretion via direct phosphorylation of GABA_A_R by protein kinase B (or Akt), a key molecule in insulin signaling that leads to the translocation of the receptors from intracellular pools to the cell surface and the subsequent membrane hyperpolarization and closure of voltage-dependent calcium channels (Xu et al., 2006).

Intra-islet insulin action has a predominant role in the regulation of glucagon secretion from the α-cells. Previous studies suggested that insulin signaling in the α-cells plays an important role in glucose-dependent glucagon secretion. In clonal glucagon-secreting α-cell lines, the ablation of the insulin receptor using siRNA techniques diminished high-glucose-induced suppression of glucagon secretion (Diao et al., 2005). Furthermore, mice lacking α-cell specific insulin receptor exhibited elevated glucagon secretion, hyperglucagonemia in the fed state, impaired glucose intolerance and hyperglycemia (Kawamori et al., 2009), indicating the inhibitory effects of intra-islet insulin on glucagon secretion (Bansal and Wang, 2008). The concept that insulin is a physiological suppressor of glucagon secretion is also supported by clinical studies. Studies involving clamp technique, which keeps glucose constant and thus allows for the effects of changes in glucose levels on glucagon secretion to be readily isolated, showed that administrations of insulin suppressed glucagon secretion in healthy humans (Raskin et al., 1975), and patients with type 1 (Raskin et al., 1976) or type 2 diabetes (Greenbaum et al., 1991; Hamaguchi et al., 1991). Similar observations were obtained in diabetic animal models (Braaten et al., 1974; Blazquez et al., 1976). Conversely, blockage of insulin signaling using insulin neutralizing antibody significantly increased glucagon levels in the perfused pancreas of rodents (Maruyama et al., 1984). At the molecular level, insulin can activate intra-islet insulin signaling involving the activation of the PI3K/Akt signaling pathway, which induces subsequent phosphorylation of the β subunit of GABA_A_R and the translocation of receptors from the intra-cellular pool to the plasma membrane. The resulting increase in the α-cell surface expression of GABA_A_Rs leads to α-cell membrane hyperpolarization and the suppression of glucagon secretion (Xu et al., 2006).

The unique arrangement of islet microvasculature is closely associated with the regulatory role of insulin on glucagon release. It has been previously demonstrated in both human and animal models that α-cells are located downstream of β-cells (Bonner-Weir and Orci, 1982; Stagner et al., 1988; Stagner and Samols, 1992). This permits a direct regulation of glucagon secretion by exposing α-cells to a high concentration of insulin. A recent study suggested that the cytoarchitecture of pancreatic islets may differ among species (Cabrera et al., 2006). Although human islets are morphologically different from those of rodent islets, such that the β-cells are located in the core and α-cells in the mantle, the human islets displayed anatomical subdivisions in which the β-cells are surrounded by α-cells (Cabrera et al., 2006; Bosco et al., 2010).

Insulin also inhibits glucagon synthesis by means of repressing the proglucagon gene (Chen et al., 1989; Philippe, 1989), presumably through the activation of the PI3K/Akt signaling pathway (Schinner et al., 2005). Several co-factors and transcription factors, including FoxO1 and Foxa2, are critical in mediating this transcriptional regulation through their direct binding to the proximal promoter region of the proglucagon gene (Philippe et al., 1995; Gauthier et al., 2002; Schinner et al., 2005; McKinnon et al., 2006). The evidence that the ablation of this *in trans* process diminishes the inhibitory effects of insulin on glucagon biosynthesis and secretion (McKinnon et al., 2006) illustrates the importance of intra-islet insulin in modulating α-cell function.

Insulin is not the sole regulator within an islet: glucagon secretion is regulated in autocrine and paracrine fashions, involving a number of islet cell secretory products including GABA produced by the β-cells (Xu et al., 2006; Braun et al., 2010), glutamate produced by the α-cells (Eto et al., 2003; Salehi et al., 2004; Uehara et al., 2004; Cabrera et al., 2006), somatostatin (Cejvan et al., 2003; Hauge-Evans et al., 2009), and possibly incretins (Gromada and Rorsman, 2004; Marchetti et al., 2012) and ghrelin (Salehi et al., 2004; Zhou et al., 2007). It should be noted that L-glutamate and GLP-1 are known to stimulate insulin secretion; thus, their inhibitory effects on glucagon may be indirect and mediated through insulin actions. Clinical evidence suggests that indirect reciprocal β-cell–mediated signaling of α-cells appears to be predominant over the direct α-cell signaling in the regulation of glucagon secretion (Banarer et al., 2002; Gosmanov et al., 2005). This notion is consistent with the physiological relevance underlying glucagon secretion. For instance, under euglycemic basal conditions, β-cell secretory products tonically restrain α-cell glucagon secretion; under β-cell stimulatory conditions, i.e., after meal ingestion, an increase in β-cell secretion counteracts the direct α-cell stimulation, leading to no change or suppression of glucagon secretion from the α-cells (Cooperberg and Cryer, 2009). However, in hypoglycemia, a decrease in β-cell secretion, in concert with a low α-cell glucose concentration, stimulates α-cell glucagon secretion (Barg et al., 2000; Bevan, 2001; Banarer et al., 2002; Bancila et al., 2005). This regulatory mechanism is further supported by a recent study indicating that an increase in insulin *per se* suppresses glucagon secretion and a decrease in insulin *per se*, in concert with a low glucose concentration, stimulates glucagon secretion in humans (Cooperberg and Cryer, 2010).

Interestingly, insulin coordinates with GABA to suppress α-cell secretion via α-cell membrane hyperpolarization, which inhibits the exocytotic machinery (Xu et al., 2006). In contrast, when this cooperation occurs in β-cells, it enhances β-cell secretion in a fine-tuned range (Bansal et al., 2011). GABA, a non-coding amino acid produced by β-cells, induces membrane hyperpolarization of α-cells (Xu et al., 2006), whereas in the β-cells, it exerts depolarizing trophic effects (Soltani et al., 2011; Braun et al., 2010). Furthermore, the glutamate released from the α-cells can act upon its own cells, although controversial (Uehara et al., 2004), to potentiate (Cabrera et al., 2008) its own secretory ability in an autocrine fashion.

**Figure 1:**
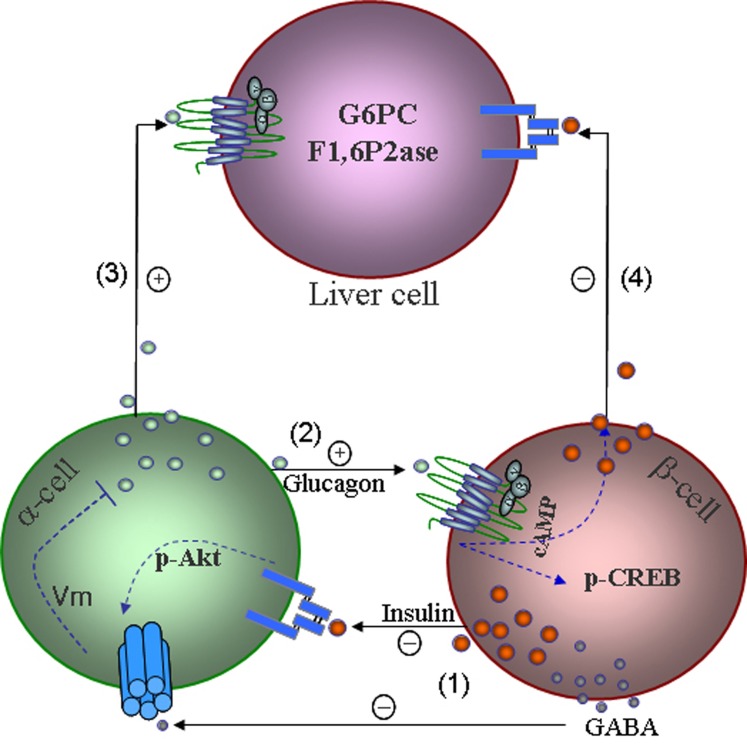
**A model shows mechanisms underlying intra-islet hormonal regulation and their impact on liver glucose production.** (1) Insulin, incorporation with GABA produced from β-cells suppresses glucagon secretion via membrane hyperpolarization. (2) Glucagon stimulates glucose-competent β-cell secretion and increases α-cell gene transcription via activation of CREB. (3) Glucagon increases its action in the liver on glucose production under glucagon stimulatory conditions. (4) Insulin increases the suppressive effect of insulin on hepatic glucose production by reducing gluconeogenesis and glycogenolysis. Insulin suppresses glucagon secretion via modulating K_ATP_ channels and repressing the proglucagon gene, as well as the effects of glucagon and insulin on a variety of organs and tissues are not shown.

Activation of α-cell insulin receptor stimulates GABA_A_R phosphorylation at the β3 subunit, enhancing cell surface expression of the GABA_A_R and leading to α-cell hyperpolarization and subsequent suppression of glucagon secretion (Xu et al., 2006; Bansal and Wang, 2008) (Figure 1). In cultured clonal α-cells, GABA_A_R insertion into the plasma membrane is mediated by insulin signaling involving the activation of the PI3K/Akt signaling pathway. In isolated rat islets, treatment with glucose suppressed glucagon secretion, as a result of enhanced intra-islet insulin action on the α-cells; insulin signaling blockage in α-cells diminishes glucose-induced suppression of glucagon secretion (Xu et al., 2006). Therefore, the intra-islet insulin-Akt- GABA_A_R pathway is critical in the regulation of glucagon secretion and maintaining an appropriate insulin-to-glucagon ratio (Xu et al., 2006), which is essential for keeping the blood glucagon within a normal range. Remarkably, in a cellular model of “insulin resistance,” where high concentrations of glucose and insulin are exposed to α-cells to mimic hyperglycemia and hyperinsulinemia, subsequent applications of insulin fail to increase GABA_A_R on the cell surface and fail to inhibit glucagon secretion (Xu et al., 2006). These findings provide a molecular mechanism by which glucose-induced suppression of glucagon secretion is mediated by the intra-islet insulin-Akt-GABA_A_R pathway (Figure 1). Defects in this signaling pathway, referred to as α-cell insulin resistance (Larsson and Ahren, 2000; Xu et al., 2006), appear to be a major contributor to hyperglucagonemia and hyperglycemia in type 2 diabetes. Evidence supporting this notion is consistent with clinical observations that α-cell insulin resistance exaggerates glucagon responses to stimuli in type 2 diabetic patients (Tsuchiyama et al., 2007). This pathway may also provide a mechanistic explanation, at least in part, for hypoglycemia occurring in insulin-treated type 1 diabetic patients. The α-cells of these patients, due to a lack of suppression by endogenous insulin, were rendered hypersensitive to exogenous insulin (Bansal and Wang, 2008).

## Mechanisms of glucagon action

The mechanism underlying the regulation of α-cell mass expansion and glucagon in response to metabolic changes has not been extensively studied. Observations from a recent study using db/db mice demonstrated that α-cell mass increased during the development of insulin resistance and hyperinsulinemia (Liu et al., 2011). The expansion of α-cell mass under insulin resistant conditions is likely a direct adaptive response to compensate increased demand for insulin in these diabetic mice. This is consistent with the observations that lacking α-cell mass expansion and glucagon secretion may cause secondary inability of the β-cell mass to adapt peripheral insulin resistance in mice under excessive nutrient feeding conditions (Ellingsgaard et al., 2008). Thus it is suggestive that, the intra-islet glucagon plays an important role in the maintenance of β-cell mass homeostasis and β-cell competence. This notion is consistent with the findings that treatment of glucagon potentiates glucose-induced insulin release in isolated adult human islets (Huypens et al., 2000). The perception is further supported by the evidence that transgenic murine pancreatic β-cells specifically overexpressing GCGR displayed enhanced glucose-competent insulin secretion, associated with increased β-cell mass and pancreatic insulin content, rendering the mice partially protected from high-fat diet feeding-induced hyperglycemia and impaired glucose tolerance (Gelling et al., 2009). Conversely, impaired β-cell function exemplified by declined glucose-induced insulin in isolated islets were profound in Gcgr^−/−^ mice lacking glucagon actions (Sorensen et al., 2006). Furthermore, pancreas perfusion studies using specific GCGR antagonists suggested that the intra-islet glucagon-induced trophic effects on the β-cells are mainly through GCGR rather than GLP-1 receptors on islet β-cells (Kawai et al., 1995), although the gut-derived insulinotropic hormone is recently found to be also produced by the α-cells and exerts local actions (Marchetti et al., 2012). The intra-islet trophic effects of glucagon have been further studied using transgenic mice expressing GCGR under control of the muscle specific creatine kinase (Mck) promoter (Maharaj et al., 2012). The Mck/Gcgr mice, which displayed mild hyperglucagonemia (but unchanged circulating GLP-1 levels), are euglycemic under basal conditions but are resistant to streptozotocin-induced β-cell injury partially due to enhanced intra-islet action of both glucagon and insulin on β-cell competence (Maharaj et al., 2012).

## Role of glucagon in diabetes

Chronic hyperglucagonemia is a major contributor to the development of diabetic hyperglycemia, due to excessive hepatic glucose output either under basal or postprandial conditions (Jiang and Zhang, 2003). Therefore, a strategy involving neutralizing peripheral glucagon actions may be beneficial for hyperglycemia in diabetes. This notion has been tested in recent studies using antibodies antagonizing GCGR, and it has been shown that administration of monoclonal antibodies normalized blood glucose levels in obese diabetic mice and improved glucose tolerance in normal mice and monkeys (Gu et al., 2009; Yan et al., 2009). Various approaches have been reported that are effective in attenuating glucagon actions, including the use of GCGR antagonists and GCGR antisense oligonucleotides (ASOs). Clinical studies showed that GCGR antagonism significantly reduced hyperglucagonemic stimuli-induced hyperglycemia in humans (Petersen and Sullivan, 2001). Consistently, reduction in GCGR expression using ASOs significantly decreased blood glucose, circulating triglyceride, free fatty acids, and improved glucose tolerance by diminishing glucagon actions in type 2 diabetes prone db/db mice (Liang et al., 2004). The effects of ablation of glucagon actions on the development of insulin-deficient diabetes have been studied in glucagon receptor knockout mice (Gcgr^−/−^). Despite hyperglucagonemia, as a consequence of pancreatic α-cell hyperplasia and excessive glucagon production, Gcgr^−/−^ mice are hypoglycemic (Gelling et al., 2003). Interestingly, ablation of the β-cells using high-dose streptozotocin, though causing severe hyperglycemia and hyperketonemia in the wildtype littermates, did not cause hyperglycemia or laboratory manifestations of diabetes in these knockout mice, indicating that ablating Gcgr prevents insulin-deficient type 1 diabetes in mice (Lee et al., 2011). The critical role of glucagon in the maintenance of glucose homeostasis had been further illustrated in the Gcgr^−/−^ mice when adenoviral restoration of hepatic GCGR expression consented to the occurrence of diabetes in these mice after streptozotocin destroyed their β-cells (Lee et al., 2012), suggesting that hepatic glucagon suppression is a key therapeutic target in diabetes.

Glucagon plays a pivotal role in maintaining functions of various organs and tissues. Although ablation of hepatic glucagon actions can prevent the occurrence of diabetic hyperglycemia and metabolic manifestations following β-cell destruction (Lee et al., 2011, 2012), complete elimination of glucagon action can produce adverse effects, which are exemplified by the phenotypes of Gcgr^−/−^ mice who have increased fetal lethality, defective islet development and impaired glucose competence in the β-cells (Sorensen et al., 2006; Vuguin et al., 2006). To investigate the effects of attenuating hepatic glucagon actions while enhancing intra-islet glucagon actions, our laboratory has generated Mck/Gcgr mice, a line with muscle-specifically expressing GCGR (Maharaj et al., 2012). The rationale behind this approach is that the ectopic overexpression of GCGR in the skeletal muscle, where little GCGRs are produced endogenously, creates a decoy receptor to neutralize excess circulating glucagon and elevates intra-islet glucagon action as a consequence of α-cell compensation to the trapping of glucagon in the muscle in Mck/Gcgr mice (Maharaj et al., 2012). These transgenic mice displayed a significant decrease in hepatic glucose-6-phosphatase and fructose-1,6-bisphosphatase mRNA levels, suggesting a reduction in gluconeogenesis and glycogenolysis. Remarkably, the Mck/Gcgr mice are euglycemic and possess higher capability in maintaining glycemic stability, particularly under extreme conditions of glucose influx or efflux exemplified by β-cell injury (Maharaj et al., 2012) and excessive nutrients feeding (Maharaj and Wang unpublished data). The reduced liver gluconeogenesis, glycogenolysis and elevated intra-islet glucagon action as a consequence of glucagon trapping and α-cell compensation, respectively, suggest that neutralizing peripheral glucagon actions, while enhancing intra-islet glucagon actions, may present a useful therapeutic approach for diabetes.

## Summary

Glucose is a primary determinant of glucagon secretion by α-cells. Within an islet, glucose-stimulated insulin secretion acts as a potent primary physiological regulator to suppress glucagon secretion through a mechanism involving the activation of the insulin-GABA signaling pathway, modulation of K_ATP_ activity and suppression of proglucagon gene expression. The regulation of glucagon secretion is multifaceted, and the process of regulating glucagon secretion involves other factors including the autonomic nervous system, somatostatin, ion channels, GLP-1 and L-glutamate. It should be noted that L-glutamate and GLP-1 are known to stimulate insulin secretion; thus, their inhibitory effects on glucagon may be indirect and mediated through insulin actions. Glucagon plays a pivotal role in protecting the body against hypoglycemia through enhanced hepatic output by stimulating glycogenolysis and gluconeogenesis while inhibiting glycolysis and glycogenesis. Intra-islet paracrine glucagon action on modulation of β-cell function is important in the maintenance of β-cell glucose competence, particularly under extreme conditions of glucose influx and efflux. Therefore, an approach that suppresses liver glucose production while enhancing intra-islet glucagon actions may present a new therapeutic strategy for diabetes.

### Conflict of interest statement

The authors declare that the research was conducted in the absence of any commercial or financial relationships that could be construed as a potential conflict of interest.
